# Anti-Epileptic Activity of Mitocurcumin in a Zebrafish–Pentylenetetrazole (PTZ) Epilepsy Model

**DOI:** 10.3390/ph17121611

**Published:** 2024-11-29

**Authors:** Alin Dumitru Ciubotaru, Carmen-Ecaterina Leferman, Bogdan-Emilian Ignat, Anton Knieling, Delia Lidia Salaru, Dana Mihaela Turliuc, Liliana Georgeta Foia, Lorena Dima, Bogdan Minea, Luminita Diana Hritcu, Bogdan Ionel Cioroiu, Laura Stoica, Ioan-Adrian Ciureanu, Alin Stelian Ciobica, Bogdan Alexandru Stoica, Cristina Mihaela Ghiciuc

**Affiliations:** 1 Discipline of Pharmacology, Faculty of Medicine, “Grigore T. Popa” University of Medicine and Pharmacy Iasi, 16 Universitatii Street, 700115 Iasi, Romania; carmen-ecaterina.leferman@umfiasi.ro (C.-E.L.); cristina.ghiciuc@umfiasi.ro (C.M.G.); 2 Discipline of Biochemistry, Faculty of Medicine, “Grigore T. Popa” University of Medicine and Pharmacy, 16 Universitatii Street, 700115 Iasi, Romania; 3 Neurology Department, Clinical Rehabilitation Hospital, 14 Pantelimon Halipa Street, 700661 Iasi, Romania; emilian.ignat@umfiasi.ro; 4 Discipline of Neurology, Faculty of Medicine, “Grigore T. Popa” University of Medicine and Pharmacy, 16 Universitatii Street, 700115 Iasi, Romania; 5 Discipline of Forensic Medicine, Faculty of Medicine, “Grigore T. Popa” University of Medicine and Pharmacy Iasi, 16 Universitatii Street, 700115 Iasi, Romania; anton.knieling@umfiasi.ro; 6 Institute of Forensic Medicine, 4 Buna Vestire Street, 700455 Iasi, Romania; 7 Institute of Cardiovascular Diseases, 50 Carol I Avenue, 700503 Iasi, Romania; deliasalaru@gmail.com; 8 Discipline of Neurosurgery, Faculty of Medicine, “Grigore T. Popa” University of Medicine and Pharmacy, 16 Universitatii Street, 700115 Iasi, Romania; mihaela.turliuc@umfiasi.ro; 9 “Prof. Dr. N. Oblu” Emergency Clinical Hospital, 2 Ateneului Street, 700309 Iasi, Romania; 10 Discipline of Biochemistry, Faculty of Dental Medicine, “Grigore T. Popa” University of Medicine and Pharmacy Iasi, 16 Universitatii Street, 700115 Iasi, Romania; georgeta.foia@umfiasi.ro (L.G.F.); bogdan-minea@umfiasi.ro (B.M.); 11 Department of Fundamental Disciplines and Clinical Prevention, Faculty of Medicine, Transylvania University of Brasov, 59 Nicolae Balcescu Street, 500019 Brasov, Romania; lorena.dima@unitbv.ro; 12 Internal Medicine Clinic, “Ion Ionescu de la Brad” University of Life Sciences, 3 Mihail Sadoveanu Alley, 700490 Iasi, Romania; lumidih@yahoo.com; 13 Research Center for Oenology, Romanian Academy, Iasi Branch, 9 Mihail Sadoveanu Alley, 700490 Iasi, Romania; bogdan.cioroiu@acadiasi.ro; 14 Discipline of Cell and Molecular Biology, “Grigore T. Popa” University of Medicine and Pharmacy, 16 Universitatii Street, 700115 Iasi, Romania; laurastoica2004@yahoo.com; 15 Department of Medical Informatics and Biostatistics, “Grigore T. Popa” University of Medicine and Pharmacy, 16 Universitatii Street, 700115 Iasi, Romania; adrian.ciureanu@umfiasi.ro; 16 Department of Biology, Faculty of Biology, “Alexandru Ioan Cuza” University of Iasi, 20A Carol I Avenue, 700505 Iasi, Romania; alin.ciobica@uaic.ro; 17 Center of Biomedical Research, Romanian Academy, Iasi Branch, 2 Teodor Codrescu Street, 700481 Iasi, Romania; 18 St. Mary’s Emergency Children Hospital, 62 Vasile Lupu Street, 700309 Iasi, Romania

**Keywords:** epilepsy, antiseizure medications, mitocurcumin, curcumin, valproate, zebrafish, PTZ, locomotor activity, seizure, drug-resistant epilepsy

## Abstract

**Background/Objectives**: Ongoing challenges in epilepsy therapy warrant research on alternative treatments that offer improved efficacy and reduced side effects. Designed to enhance mitochondrial targeting and increase bioavailability, mitocurcumin (MitoCur) was evaluated for the first time as an antiepileptic agent, with curcumin (Cur) and sodium valproate (VPA), a standard antiepileptic drug, included for comparison. This study investigated the effects on seizure onset, severity, and progression in a zebrafish model of pentylenetetrazole (PTZ)-induced seizures and measured the concentrations of the compounds in brain tissue. **Methods**: Zebrafish were pre-treated with MitoCur and Cur (both at 0.25 and 0.5 µM doses) and VPA (0.25 and 0.5 mM) and observed for four minutes to establish baseline locomotor behavior. Subsequently, the animals were exposed to a 5 mM PTZ solution for 10 min, during which seizure progression was observed and scored as follows: 1—increased swimming; 2—burst swimming, left and right movements; 3—circular movements; 4—clonic seizure-like behavior; 5—loss of body posture. The studied compounds were quantified in brain tissue through HPLC and LC-MS. **Results:** Compared to the control group, all treatments reduced the distance moved and the average velocity, without significant differences between compounds or doses. During PTZ exposure, seizure latencies revealed that all treatments effectively delayed seizure onset up to score 4, demonstrating efficacy in managing moderate seizure activity. Notably, MitoCur also provided significant protection against the most severe seizure score (score 5). Brain tissue uptake analysis indicated that MitoCur achieved higher concentrations in the brain compared to Cur, at both doses. **Conclusions:** These results highlight the potential of MitoCur as a candidate for seizure management.

## 1. Introduction

Epilepsy remains one of the most prevalent and complex neurological disorders, presenting a significant global health challenge [1]. The multifaceted nature of epilepsy [2], coupled with its diverse etiological factors and clinical manifestations [3,4,5,6,7], necessitates a comprehensive approach to its understanding and treatment [8]. Despite advancements in neurology and pharmacology, the management of epilepsy continues to confront substantial hurdles, highlighting the urgent need for innovative research and therapeutic strategies [9,10].

The mechanisms underlying the transition from normal brain function to seizure activity are still unclear [11]. The disorder encompasses a broad spectrum of seizure types and syndromes, each defined by unique characteristics and underlying mechanisms. The variability in seizure presentation and severity underscores the complexity of epilepsy as a clinical and research domain [12].

Despite a vast potential market for CNS drugs, most fail to reach commercialization because of inefficient delivery methods [13]. The blood–brain barrier (BBB) poses a major obstacle, with its tight junctions permitting only selective passage, which maintains homeostasis and protects against toxins, but also restricts drug access [14]. Current CNS treatments typically offer only symptomatic relief and do not halt disease progression or repair cellular damage, signaling a clear need for innovative drug delivery systems [15].

While the arsenal of antiepileptic drugs (AEDs) has expanded over the years, a significant proportion of patients continue to experience refractory epilepsy, with seizures that are not adequately controlled by existing medications [16]. Furthermore, classical AEDs are often associated with side effects, such as sedation, cognitive impairment, and hepatotoxicity. These aspects represent substantial challenges, underscoring the limitations of current treatments and the critical need for novel therapeutic approaches with enhanced efficacy and safety profiles.

Curcumin (Cur), a natural compound derived from turmeric and commonly used as a dietary supplement, is known for its capacity to fight inflammation, oxidative stress [17], and neuronal damage [18], proving to be a promising agent in epilepsy research conducted in laboratory settings [19]. Its effectiveness in preventing seizures across various experimental frameworks, including both chemically and electrically induced models, has been documented [20]. Particularly, it has been effective in diminishing the severity and frequency of seizures in models involving kainate to simulate temporal lobe epilepsy [21]. While its protective effects against seizures are likely due to its anti-inflammatory and antioxidative properties, detailed mechanisms are yet to be fully understood.

A significant limitation in the therapeutic use of Cur is its poor bioavailability and reduced brain penetration, which has led to the exploration of nanoliposome and liposome methods to improve its delivery to the brain and overall efficacy [22]. Thus, Cur formulations, such as those encapsulated in nanoparticles or reduced to a micronized state, have been successful in mitigating cognitive decline and neuronal damage in epilepsy models triggered by PTZ [23,24]. Moreover, researchers have proposed various strategies to enhance the delivery of CNS agents, including receptor-mediated endocytosis and transcellular transport, among others. These approaches aim to bypass the BBB’s restrictive nature, allowing more effective transport of therapeutic agents into the brain [25]. Nonetheless, the challenge of the poor bioavailability of Cur remains, limiting its anticonvulsant potential, and highlighting the critical need for continued research into enhancing its uptake and therapeutic benefits.

Mitocurcumin (MitoCur), a novel derivative of the naturally occurring compound Cur, has gained attention for its potential therapeutic benefits. It consists of Cur conjugated with triphenylphosphonium (TPP). Compared to simple Cur, it has significant advantages in terms of stability and water solubility, which could contribute to enhanced therapeutic efficacy [26,27]. The positive charges of the TPP groups enhance membrane crossing and mitochondrial targeting, which could represent an important advantage of MitoCur, given the critical role of mitochondrial dysfunction in epileptogenesis and seizure activity.

The zebrafish (*Danio rerio*) has emerged as a powerful model organism in biomedical research. This model offers distinct advantages due to its unique combination of genetic and physiological similarity to humans, and practical advantages for modeling epilepsy. Zebrafish share many homologous brain structures with humans, such as the habenula, cerebellum, and hypothalamus, as well as highly conserved neurotransmitter systems, making them a relevant model for neurological disorders. Zebrafish membranes are permeable to drugs in the bathing medium, enabling simple administration and real-time observation of seizure activity through behavioral, molecular, and electrophysiological analyses. Furthermore, zebrafish experience seizure activity that replicates many key aspects of mammalian seizures, offering valuable insights into drug absorption, distribution, metabolism, excretion, and toxicity (ADME). These features, combined with their suitability for high-throughput screening, make zebrafish an ideal model for studying epilepsy and evaluating potential antiepileptic therapies [28,29].

The pentylenetetrazole (PTZ)-induced seizure model in zebrafish has been extensively utilized to mimic epileptic activity [30]. PTZ acts as a GABA(A) receptor antagonist, reducing inhibitory neurotransmission, which closely mirrors the neuronal hyperexcitability observed in human epilepsy. This model is widely recognized for inducing acute seizures that replicate key features of human epileptic activity, including generalized tonic–clonic seizures and distinct stages of seizure progression.

This study aims to investigate the antiseizure potential of MitoCur in the PTZ-induced zebrafish model of epilepsy, by comparing its efficacy with sodium valproate (VPA), a traditional antiepileptic drug, and with Cur, and by examining its effects on behavioral changes and seizure dynamics. We hypothesized that MitoCur would exhibit anticonvulsant properties, mediated through mitochondrial modulation, and would offer a novel therapeutic strategy for epilepsy management.

## 2. Results

### 2.1. Behavioral Assessment—Locomotor and Exploratory Behavior

**Figure 1:**
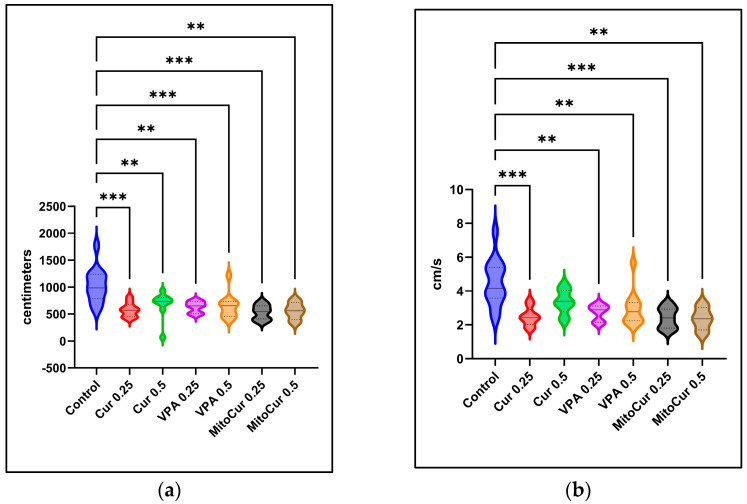
The effects of antiepileptic treatments on the locomotor and exploratory behavior of the zebrafish over a 4-min observation period, prior to seizure inducing agent exposure—violin plots illustrating the distribution of the values, the median (continuous line), and the interquartile range (dotted lines): (**a**) Total distance moved (cm); (**b**) Average velocity (cm/s). Statistical significance is indicated as ** *p* < 0.01, *** *p* < 0.001 (ordinary one-way ANOVA + Tukey’s multiple comparisons test). The *p* values were adjusted to account for multiple comparisons.

Movement data were analyzed by comparing total distances moved and average velocity across treatment groups. Results from one-way ANOVA, followed by Tukey’s multiple comparisons test, indicated that all treatments significantly reduced the total distances moved (Figure 1a) compared to the control group. All treatments, except Cur 0.5, also reduced the average velocity (Figure 1b) compared to the control group. Although the differences between Cur 0.5 and the control did not reach statistical significance (*p* = 0.065), this treatment showed the same trend of reducing locomotor activity.

### 2.2. Behavioral Assessment—PTZ-Induced Seizures

**Figure 2:**
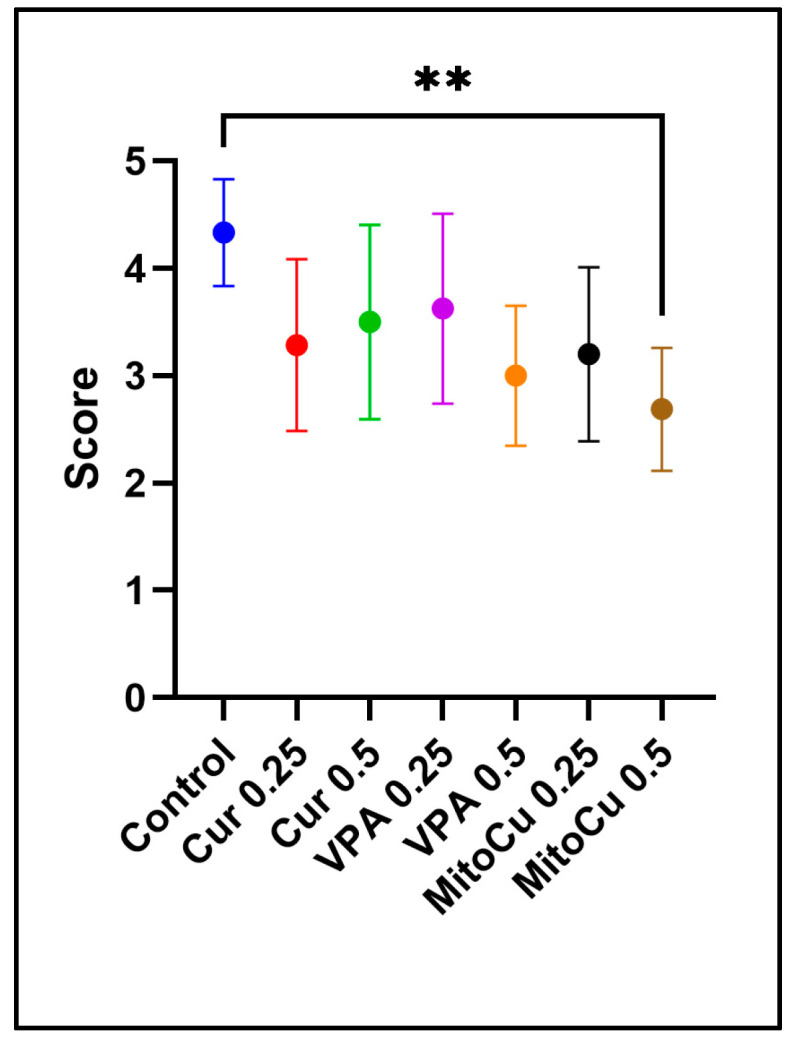
Average final seizure scores after 10 min (mean with 95% confidence interval). MitoCur at 0.5 μM significantly reduced seizure scores compared to the control group (*p* = 0.0025). Statistical significance is indicated as ** *p* < 0.01 (Kruskal–Wallis test + Dunn’s multiple comparisons test). The *p* values were adjusted to account for multiple comparisons.

The final seizure scores of zebrafish at the end of a 10-min observation period were compared by performing the Kruskal–Wallis test, followed by Dunn’s multiple comparisons test. The data showed that all treatments tended to reduce the scores reached after 10 min compared to the control group, but only MitoCur 0.5 reached statistical significance (*p* = 0.0025) (Figure 2).

**Figure 3:**
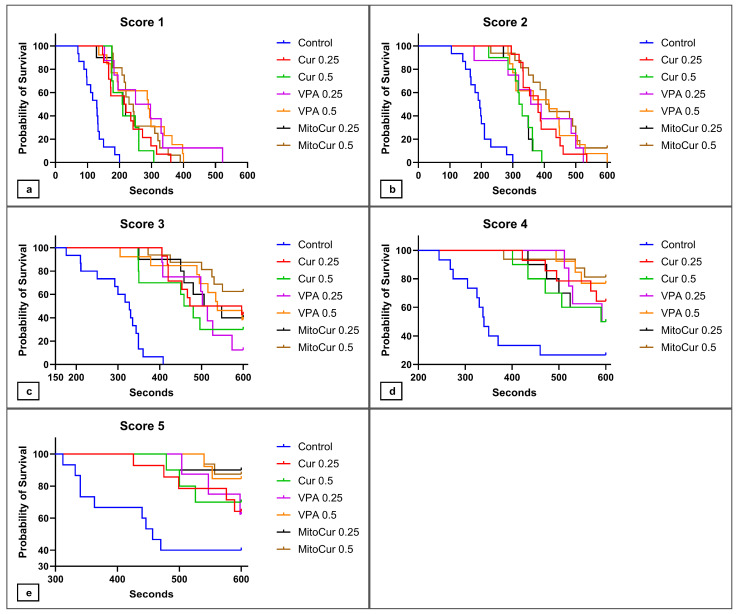
Kaplan–Meier survival curves illustrating the probability of zebrafish not reaching different seizure scores after anti-epileptic treatment followed by PTZ exposure: (**a**) probability of not reaching score 1; (**b**) probability of not reaching score 2; (**c**) probability of not reaching score 3; (**d**) probability of not reaching score 4; (**e**) probability of not reaching score 5. The Gehan–Breslow–Wilcoxon test was used to compare the curves. To compare curves two-by-two while accounting for multiple comparisons, the new significance threshold for the *p* values (*p* < 0.0024) was calculated by using the Bonferroni method.

The latencies to reach the different seizure scores were analyzed by comparing Kaplan–Meier survival curves, where the event of interest was reaching a certain seizure score in the PTZ-induced seizure model (Figure 3). As the positive outcome in the analysis of latencies could be defined as preventing or delaying as much as possible the progression towards a seizure, we deemed the time it took the fish to reach a certain score as an important aspect to consider. We therefore compared the survival curves by using the Gehan–Breslow–Wilcoxon test, which places more weight on events of interest that occur at early time points. The Bonferroni method was used to adjust for multiple comparisons (significance at *p* < 0.0024).

Regarding latencies to seizure score 1 (Figure 3a), all treatments—Cur 0.25 (*p* < 0.0001), Cur 0.5 (*p* = 0.0002), VPA 0.25 (*p* = 0.0005), VPA 0.5 mM (*p* < 0.0001), MitoCur 0.25 (*p* = 0.0007), and MitoCur 0.5 (*p* < 0.0001)—significantly delayed the onset of seizures compared to the control group. These results indicate comparable antiseizure efficacy across treatments.

For seizure score 2 (Figure 3b), all treatments significantly delayed the progression to score 2 compared to the control (*p* < 0.0001). Moreover, MitoCur 0.5 prevented the onset of score 2 in the case of two animals. This is probably why MitoCur 0.5 significantly delayed the onset of score 2 compared to Cur 0.25 (*p* = 0.0022) and Cur 0.5 (*p* = 0.0022) as well, highlighting the potent antiseizure effects of MitoCur.

The analysis of latencies to seizure score 3 (Figure 3c) revealed that all treatments significantly delayed seizure progression compared to the control group (*p* ≤ 0.0002). In the case of certain animals, the tested compounds even prevented the onset of score 3, demonstrating their protective effects against PTZ-induced seizures.

All treatments showed a tendency to delay the onset of seizure score 4 (Figure 3d), but only Cur 0.25 (*p* = 0.0018), VPA 0.5 (*p* = 0.0010), MitoCur 0.5 (*p* = 0.0002) significantly reduced the probability of reaching seizure score 4.

In the case of seizure score 5 (Figure 3e), the same tendency to delay seizure progression was displayed by all treatments, but only MitoCur 0.5 reached statistical significance (*p* = 0.0017), compared to the control group. These results highlight the strong anticonvulsant potential of mitocurcumin.

### 2.3. Compound Detection and Quantification in Brain Tissue by HPLC and LC-MS

**Figure 4:**
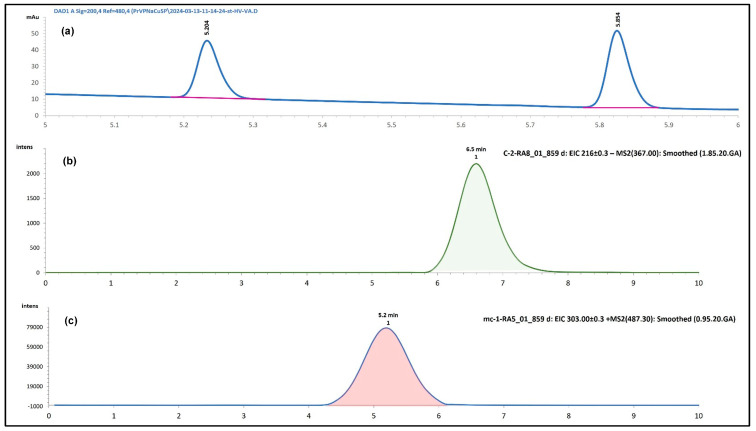
Chromatograms: (**a**) Heptanoic acid (λ = 200 nm) (Rt = 5.234), Sodium valproate (λ = 200 nm) (Rt = 5.585); (**b**) Mitocurcumin (EIC + MS2(487.30) → 303.00), Rt = 6.5; (**c**) Curcumin (EIC − MS2(367.00) → 216.80), Rt = 5.0).

The analytical method used for detecting VPA (Figure 4a), Cur (Figure 4b), and MitoCur (Figure 4c) was validated for accuracy, precision, and reproducibility, and was adapted after Ramalingam et al. [31] with slight modifications. HPLC successfully quantified VPA and demonstrated high sensitivity.

Due to the limitations of HPLC in detecting Cur and MitoCur at low concentrations, the LC-MS method was employed, which was developed and validated after Ramalingam et al. [31].

**Figure 5:**
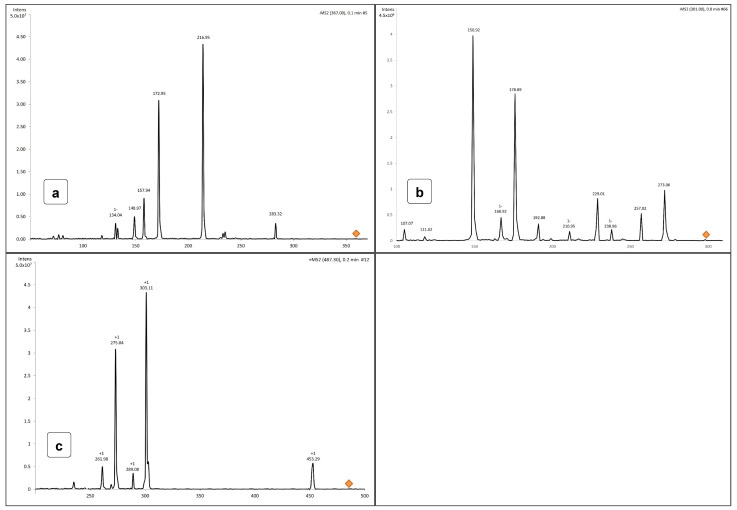
Molecular spectra for (**a**) Cur (EIC − MS2 (367.0 → 216.80), (**b**) Quercetin (EIC − MS2 (301.0 *m*/*z* → 178.89 *m*/*z*), and (**c**) MitoCur (EIC + MS2(487.30) → 303.00).

For Quercetin and Cur, the negative ionization mode was used, with the following mass spectrum characteristics: Cur—molecular ion at *m*/*z* 367.0 and fragments at 216.95, 172.95, and 157.94 (Figure 5a)—negative ionization); Quercetin—molecular ion at *m*/*z* 301.0 and fragments at 273.06, 178.89, and 150.92 (Figure 5b)—negative ionization.

With a molecular mass of 1044, MitoCur exhibits multiple ionizations. Positive ionization mode was used, and the molecular mass without chloride ions was 973.0. The molecular ion at [M + H]^2+^ had a calculated mass of 486.5. Determined molecular ion was 487.30 (δ = −0.2). The following fragments were identified for MitoCur: 453.29, 303.11, 275.04, 289.08, and 261.98 (Figure 5c).

The method demonstrated excellent linearity, with correlation coefficients of 1.000 for Cur, 0.998 for MitoCur, and 0.999 for VPA. A higher slope was exhibited by Cur (31.64), compared to MitoCur (1.459) and VPA (1.180).

The LOQ and LOD values demonstrated high sensitivity, with Cur showing the lowest values for LOQ of 2.09 ± 0.44 ppb and for LOD of 0.68 ± 0.44 ppb. These results are comparable in sensitivity to findings reported in previously published methods, where the mean LOQ for Cur was 2.0 ± 0.5 ppb, and the mean LOD was 0.61 ± 0.41 ppb [31,32,33].

Instead, MitoCur had higher values of 6.92 ± 0.35 ppb for LOQ and 2.28 ± 0.12 ppb for LOD. However, an evaluation of MitoCur with reference data was not possible due to the absence of existing results, highlighting the novelty of this approach.

The HPLC quantification of VPA showed a medium value for slope of 2.37, an r^2^ value of 0.998, an LOQ of 5.71 ± 0.38 ppm, and an LOD of 3.77 ± 0.25 ppm, consistent with previous findings. Various sensitivities, expressed as LODs and LOQs, were selected based on the specificity of the instrumental methods used for comparison with the current results. Data comparisons were made in light of the analytical techniques employed, referencing the findings which had mean values for LOQ of 1.47 ± 0.54 ppm and LOD of 0.44 ± 0.15 ppm [34,35,36].

**Figure 6:**
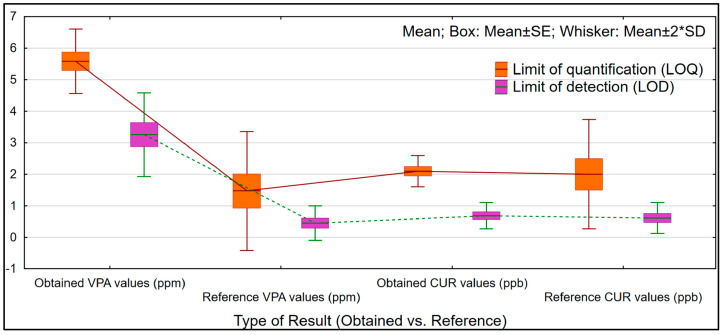
Comparison of limits of detection and limits of quantification for Cur (Reference values—Ramalingam et al. [31], Kunati et al. [32], and Hayun et al. [33]) and VPA (Reference values—Vancea et al. [34], Hara et al. [35], and Gao et al. [36]).

A comparison of the limits of quantification (LOQ) and detection (LOD) for VPA, analyzed using High-Performance Liquid Chromatography (HPLC), and Cur, analyzed using Liquid Chromatography–Mass Spectrometry (LC-MS), was conducted to clearly assess the performance of each method and presented in Figure 6.

Sample preparation involved several steps as solid liquid extraction, solvent evaporation, and residue reconstitution, and was modified after Eskandari et al. [37] for supplementary determination of Cur and MitoCur. Recovery rates were excellent, with VPA showing a recovery of 102.18%, Cur at 98.71%, and MitoCur at 96.28%, validating the method’s reliability for quantitative analysis.

**Figure 7:**
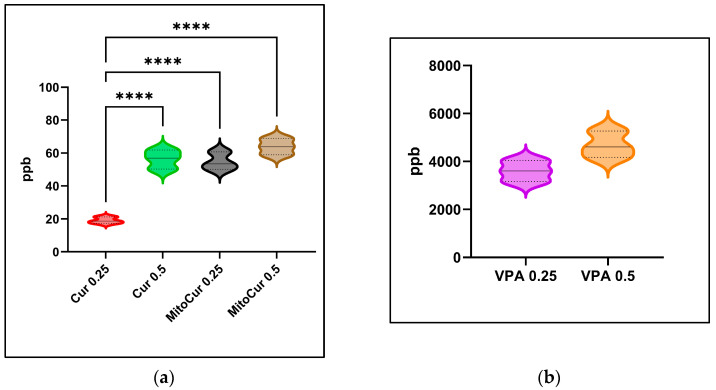
Brain concentration after 30 min of exposure by immersion—violin plots illustrating the distribution of the values, the median (continuous line) and the interquartile range (dotted lines): (**a**) Cur and MitoCur (ordinary one-way ANOVA + Tukey’s multiple comparisons test); (**b**) VPA (unpaired *t* test). Statistical significance is indicated as **** *p* < 0.0001. The *p* values were adjusted to account for multiple comparisons.

For MitoCur 0.25, the concentration in brain tissue (Figure 7a) was 54.77 ± 5.42 ppb with a relative standard deviation (RSD) of 9.89%; while, in the case of MitoCur 0.5 µM, it increased to 63.90 ± 4.95 ppb with an RSD of 7.75%. Curcumin at 0.25 µM reached a concentration of 19.11 ± 1.99 ppb with an RSD of 10.39%; at 0.5 µM, the concentration rose to 56.30 ± 5.76 ppb with an RSD of 10.23%.

Statistical analysis showed the differences to be significant between Cur 0.25 and Cur 0.5 (*p* < 0.0001), as well as between Cur 0.25 and both MitoCur 0.25 (*p* < 0.0001) and MitoCur 0.5 (*p* < 0.0001).

At a dose of 0.5 mM, VPA reached a brain concentration of 4.68 ± 0.55 ppm, with an RSD of 11.84% (Figure 7b). At 0.25 mM, the concentration was 3.60 ± 0.44 ppm, with an RSD of 12.13%. All concentrations are expressed as a mean ± SD. The retention times (RT) for both concentrations remained consistent, ranging from 5.232 to 5.893 min, confirming the method’s stability and reproducibility.

Therefore, the developed HPLC and LC-MS protocols successfully quantified VPA, Cur, and MitoCur with high accuracy and precision. Statistically significant differences in Cur and MitoCur brain concentrations were observed between the compounds and between doses, particularly highlighting significant differences between Cur and MitoCur across doses with adjusted *p*-values of less than 0.0001.

## 3. Discussion

Our study explored the anticonvulsant potential of MitoCur, a novel mitochondrial-targeted Cur derivative, alongside simple Cur and VPA, a classic anti-epileptic drug. This approach aimed to assess the therapeutic benefits of MitoCur and explore its potential role in targeting mitochondrial processes that may be relevant to epilepsy.

Both VPA [38,39] and Cur [21,24,40] have demonstrated anticonvulsant effects in rodent and zebrafish models, significantly reducing seizure frequency and severity [41,42]. These compounds are thought to act through distinct but also complementary mechanisms that contribute to seizure suppression and neuroprotection. In the case of VPA, the mechanisms of action in epilepsy primarily involve the inhibition of voltage-gated sodium channels in the cell membrane and augmentation of GABAergic activity. By increasing GABA availability, VPA stabilizes neuronal firing rates, thereby reducing excitatory neurotransmission that underlies seizure activity. Additionally, VPA has been shown to inhibit histone deacetylase (HDAC) activity, which is known to have neuroprotective and anti-inflammatory effects that may contribute to its long-term efficacy in preventing seizures and neurodegeneration [38,39].

On the other hand, Cur exerts its anticonvulsant effects via its strong antioxidant and anti-inflammatory properties, thought to mitigate the oxidative stress and neuro-inflammation often associated with epileptic seizures. Curcumin scavenges reactive oxygen species (ROS), reducing cellular stress that can otherwise lead to seizure initiation and propagation. Furthermore, Cur inhibits pro-inflammatory cytokines and modulates signaling pathways, such as nuclear factor-kappa B (NF-κB), which are implicated in neuro-inflammatory processes associated with epilepsy [21,24,40].

The limited bioavailability of Cur, however, has restricted its clinical applications, prompting research into delivery systems that enhance its therapeutic potential. Numerous studies tried to address the low bioavailability of Cur through various strategies, such as PLGA-PEG nanoparticles [43], piperine co-administration [44], nanostructured lipid carriers [45], or micronization of Cur [24]. Our study proved the anti-epileptic activity of MitoCur, a Cur modified by conjugation with two triphenylphosphonium radicals, which enhances mitochondrial targeting, improves cellular uptake and increases stability [46]. In addition, previous research on MitoCur has primarily focused on its anticancer and antibacterial properties [27,47,48,49,50], therefore its use in epilepsy models represents a novel potential application.

The doses used for both Cur and MitoCur—0.25 and 0.5 µM—were selected based on the established literature [24,51,52], and further validated through our preliminary testing to ensure efficacy in seizure modulation. Valproate was administered at doses of 0.25 mM and 0.5 mM, which are likely effective at blocking neural over-excitation and controlling behavioral seizures while minimizing adverse effects [39]. The pre-administration of the studied compounds 30 min prior to exposure to a seizure-inducing agent (PTZ) was performed to evaluate their activity in reducing seizure severity and to assess their prophylactic potential. This preemptive administration is the preferred approach in antiepileptic therapy [24,39,53]. Immersion is a widely used method in zebrafish behavioral research and is often preferred over injection techniques, as injections can cause pain and may affect the accuracy of behavioral data [54]. This method was, therefore, chosen to avoid such confounding factors in our experiment.

In zebrafish, epilepsy can be effectively induced through various methods, including chemical agents [55,56,57,58], genetic mutations [59,60,61], and optogenetic or electrical stimulation [62]. Chemical induction remains one of the most widely used approaches, providing a reliable model for studying epilepsy mechanisms and testing anticonvulsant therapies [55,58,63,64,65]. PTZ is the most widely used chemical inducer in zebrafish epilepsy research due to its significant advantages. It reliably induces rapid onset of seizure-like behaviors, such as clonic convulsions and electrographic discharges, making it a highly effective model for acute seizure studies [66,67]. The reversible nature of PTZ-induced seizures and its suitability for immersion protocols allow for consistent, high-throughput testing of potential antiepileptic compounds.

Previous studies suggest an optimal PTZ dosage range of 2.5 to 15 mM, with immersion exposure lasting 10 to 20 min [54,68,69]. In our study, we used a 5 mM concentration with a 10-min exposure, which effectively minimized mortality, enabled tracking of seizure progression, and facilitated detailed behavioral analysis, particularly in the early stages of seizure onset.

Locomotion parameters, such as distance moved and velocity, are commonly used to assess overall activity levels and provide valuable insights into the neurological and physiological states of organisms [70,71,72]. Previous research [24] has reported that Cur, and its micronized form, reduced the distance moved in adult zebrafish, with no significant difference regarding VPA. In our study, the locomotion analysis revealed that all treatments, including VPA, tended to reduce the total distances moved and velocity of zebrafish compared to the control group. The influence of MitoCur on the locomotor behavior was similar to those of Cur and VPA. Furthermore, no significant dose-dependent variation was observed. The uniformity in locomotor behavior across pretreatment groups and doses suggests that Cur, MitoCur, and VPA share similar baseline effects on motility. This consistency indicates that any observed seizure suppression post-PTZ is likely due to specific anticonvulsant properties rather than general sedative or dose-dependent effects.

Our seizure evaluation system, based on established protocols [68,69], provided a systematic approach to assess seizure manifestations in zebrafish, ranging from normal behavior to tonic–clonic seizures. This methodology allowed for a comprehensive analysis of the anticonvulsant effects of the tested compounds. Our study identified MitoCur as a promising candidate for treating generalized tonic–clonic seizures and generalized epilepsy, as it effectively reduced seizure intensity in our PTZ-induced acute model. Both Cur and VPA demonstrated protective effects in delaying the onset of moderate seizure activity (scores 3 and 4), while MitoCur at 0.5 µM exhibited the most potent anticonvulsant effect among the tested compounds, significantly lowering final seizure scores and delaying progression to all seizure stages, including the most severe (score 5). The positive results observed in our study are comparable to those reported by Bertoncello et al., where, compared to regular Cur, micronized Cur demonstrated superior anticonvulsant properties in the tonic–clonic phase and effects similar to those of VPA [24].

This superior performance of MitoCur compared to traditional Cur may be attributed to its enhanced ability to target mitochondria. Triphenylphosphonium is a widely used lipophilic cation for targeting drugs to mitochondria, and when covalently linked to a pharmacophore, it enables the specific delivery of bioactive compounds to mitochondria, achieving concentrations up to 1000-fold higher in these organelles [46].

Mitocurcumin may exert its effects by rapid modulation of oxidative pathways at the mitochondrial level, thereby reducing oxidative stress and stabilizing mitochondrial function. Furthermore, its lipophilic properties may contribute to neuronal excitability stabilization and seizure alleviation. Research has shown that other mitocans, such as MitoQ, can reduce lipid peroxidation, increase mitochondrial membrane permeability, which facilitates absorption in the brain, with applications in treating neurodegenerative diseases associated with cell death driven by oxidative stress [73]. Another mitocan, SkQ1 shows antioxidant effects at low concentrations, but becomes prooxidant at higher levels in both preclinical models and in humans. By comparison, the redox cycling of MitoQ in vivo does not overpower the body’s cellular antioxidant systems. Following preliminary exploratory tests conducted in our lab, we observed that, at the same dose, the effect of MitoCur was more pronounced than the effect of simple Cur. While MitoCur may exhibit similar antioxidant properties to Cur, it might also follow a similar pattern, where higher doses become more toxic, potentially shifting to a prooxidant effect. This could explain certain reports in the literature, which categorize MitoCur as a prooxidant under certain conditions. These characteristics of mitocans suggest the need for a detailed dose–response analysis across a broad range of concentrations to accurately define the “antioxidant window” for MitoCur, with a working hypothesis that there may be a threshold beyond which its effects become detrimental.

Interestingly, the LC-MS analysis detected that concentrations of MitoCur were higher than those of Cur at equivalent doses, suggesting potentially enhanced bioavailability or tissue retention of the mitochondria-targeted compound. This could explain the superior anticonvulsant effects of MitoCur observed in our behavioral studies and supports the hypothesis that mitochondrial targeting may improve the efficacy of Cur-based therapies.

The successful detection and quantification of these compounds at low concentrations highlight the potential of zebrafish as a model for studying the pharmacokinetics and efficacy of novel antiepileptic compounds.

MitoCur may offer, therefore, several potential advantages over traditional AEDs. Firstly, its mitochondrial targeting may allow it to address a key site of dysfunction in epileptogenesis, potentially offering a more focused and specific therapeutic mechanism. Secondly, its superior bioavailability compared to Cur, that may enable effective brain tissue concentrations at lower doses, may reduce the risk of adverse effects. Thirdly, the encouraging results regarding the prevention and delay of seizure progression documented by the behavioral tests in this study, showed that MitoCur was comparable to VPA, with some indications of protection at the most severe seizure stages.

This study has several limitations that should be acknowledged. The first one is represented by the model. While the PTZ zebrafish model effectively mimics certain aspects of epilepsy, including seizure onset and progression, it primarily reflects acute seizure activity rather than chronic epilepsy or comorbidities, such as cognitive decline or emotional disturbances. The zebrafish brain, although sharing conserved structures with humans, lacks certain features, such as a layered cortex, which may limit the direct translational relevance of some findings. Additionally, differences in metabolism and drug absorption between zebrafish and mammals could influence the pharmacokinetic profiles of the tested compounds, requiring careful extrapolation of dosing regimens to human contexts. Lastly, behavioral readouts in zebrafish, while highly sensitive and reproducible, may not capture the full spectrum of seizure-related phenotypes observed in higher-order animals or humans. Despite these limitations, the model remains a robust tool for studying the pharmacological effects of antiepileptic compounds and identifying potential therapeutic candidates. Its translational relevance lies in its reproducibility, cost-effectiveness, and ability to evaluate seizure-modulating compounds in a controlled environment, making it a valuable platform for preclinical epilepsy research.

While the findings of our study revealed encouraging results regarding MitoCur, its therapeutic profile requires further investigation, including comparisons of side effect profiles, dose–response relationships, and long-term efficacy, to fully assess its advantages and limitations relative to well-established AEDs.

For future studies, a side-by-side comparison of MitoCur with other mitocans or other antioxidant compounds [74] could provide insights into their mechanisms under oxidative stress and their potential to alleviate PTZ-induced epileptic symptoms. Observing if there are common dose-dependent effects or specific thresholds where their benefits decline might highlight potential therapeutic combinations or dosage strategies.

Another interesting study could be to investigate how each compound interacts with known antioxidants, like Vitamin E or NAC (N-Acetyl-L-Cysteine), that could clarify if they have additive, synergistic, or antagonistic effects. This could point to optimized therapeutic combinations or clarify interactions to avoid.

## 4. Materials and Methods

### 4.1. Animals

Adult wild-type zebrafish (*Danio rerio* wild-type AB strain, 4–9 months) of both sexes were obtained from a local supplier and acclimated for 4 weeks before the experiments were conducted at the “Ion Ionescu de la Brad” Iași University of Life Sciences, Iași, Romania. The animals were carefully weighted and measured to select the ones with similar weight and size (250–350 mg and 18.3–25.7 mm, respectively) to avoid putative variations of drug pharmacodynamics and pharmacokinetics. The fish were habituated to the laboratory conditions in 37 L (45 × 28 × 30 cm) aquariums (60–70 fish per aquarium). All tanks were filled with non-chlorinated water previously treated with 132 mL/L AquaSafeH (Tetra, Blacksburg, VA, USA). Group-housed fish were maintained at a temperature of 26 ± 2 °C with constant filtration and aeration of the system water under a 14:10 h light/dark cycle (light on at 8:00 a.m.; light off at 10:00 p.m.). System water pH was maintained in the 7.0–7.25 range (checked by pH strips). Fish were fed two times a day with standard feed for aquarium fish (semi-floating and not colored; 42% protein; min 4% fat; max. 3% fiber; and max. 12% ash).

All animals used in this study were experimentally naive, healthy, and free of any signs of disease. Sex was not a factor in the random choosing of fish in the experiment. The animals were tested during the light phase between 8:00 a.m. and 2:00 p.m., after a minimum 30-min acclimatization to the experimental room.

Euthanasia of animals was performed after the experiment by submersion in an ice water bath (5 parts ice/1 part water, 0–4 °C). All procedures were conducted in accordance with the European Union Directive of 22 September 2010 (2010/63/EU) and Romanian legislation concerning animal experimentations. The experimental procedures and protocols were approved by the Local Ethics Committee at the “Ion Ionescu de la Brad” Iași University of Life Sciences, Iași, Romania (license no 1760/24 October 2023). The experiment was designed to effectively implement the principles of replacement, reduction, and refinement (3Rs principle). The number of animals in each experimental group was reduced to the minimum needed to achieve consistent and reproducible results. All efforts were made to minimize animal suffering in this study.

### 4.2. Chemicals and Reagents

**Figure 8:**
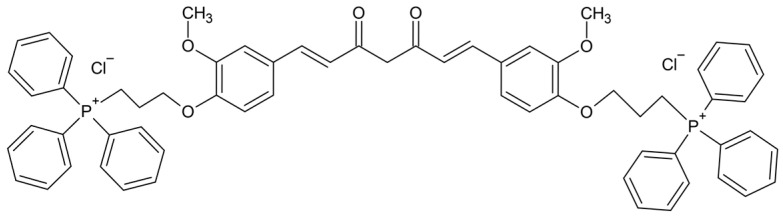
The chemical structure of the investigated mitocurcumin. The structure was performed in ChemSketch (Freeware) 2024.1.3.

Curcumin, pentylenetetrazole, and sodium valproate were purchased from Sigma-Aldrich (St. Louis, MO, USA). Mitocurcumin (1,7-Bis{3-methoxy-4-[3-(triphenylphosphonium)propoxy]-phenyl} hepta-1,6-diene-3,5-dione dichloride), illustrated in Figure 8, was purchased from Chiralsyn Laboratories (Hyderabad, India). All other reagents used were of analytical grade.

Both Cur and MitoCur were dissolved in 0.1% dimethyl sulfoxide anhydrous (DMSO) obtained from Sigma-Aldrich and diluted in the system water. Valproate was dissolved in ultrapure water while PTZ was dissolved in aquarium water. All formulations were freshly prepared.

Methanol, acetonitrile, ammonium formate, sodium acetate, and acetic acid used for HPLC investigations were of HPLC purity and were purchased from Merck (Darmstadt, Germany). The water was purified in-house using a GenPure UV-TOC ultra-pure water system (Thermo Fisher Scientific, Waltham, MA, USA) with a conductivity of 0.055~0.063 µS/cm.

### 4.3. Experiment Design

**Figure 9:**
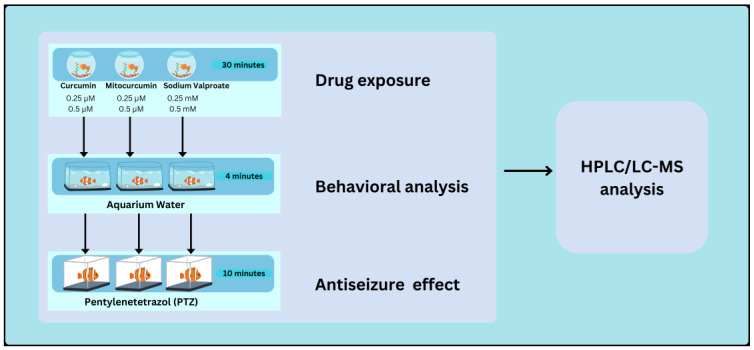
Schematic representation of the experimental design. Zebrafish were treated with three anti-epileptic compounds for 30 min, at two concentrations each: 0.25 µM and 0.5 µM for Cur and MitoCur, and 0.25 mM and 0.5 mM for VPA. Behavioral analysis was conducted in aquarium water for 4 min. The animals were subsequently exposed to PTZ (pentylenetetrazol) for 10 min, to assess the antiseizure effects of the compounds. Afterward, HPLC/LC-MS analysis was performed on brain tissue extracts to quantify drug concentrations.

The animals were exposed by immersion to two concentrations of each tested compound, for 30 min (Figure 9, Figure S1). The fish were then observed for 5 min, to determine the effects of the compounds on their behavior (Video S1). To investigate the anti-epileptic effects of the tested compounds on PTZ-induced seizures, all groups were subsequently exposed, also by immersion, to PTZ for 10 min, during which their seizure behavior was assessed (Video S2). At the end of the experiment, the animals were euthanized and the concentrations of the tested compounds in the brain were measured.

The experimental groups were structured as follows: a control group (*n* = 15), which did not receive antiepileptic treatment, and six groups exposed to 0.25 µM (*n* = 14) and 0.5 µM (*n* = 10) Cur, 0.25 µM (*n* = 10) and 0.5 µM (*n* = 16) MitoCur, as well as 0.25 mM (*n* = 8) and 0.5 mM VPA (*n* = 13), respectively. VPA was used to verify the zebrafish response to a classic AED in our PTZ-induced seizure model.

All treated animals were individually exposed to the tested compounds for 30 min in a beaker containing 0.5 L of solution, prior to the PTZ exposure. All groups were treated and analyzed in an identical manner.

The concentrations of Cur (0.25 µM and 0.5 µM) and VPA (0.25 mM and 0.5 mM) were selected based on both the existing literature [24,68,75] and preliminary studies conducted in our laboratory. Similarly, the concentrations of MitoCur (0.25 µM and 0.5 µM) were chosen in alignment with Cur doses and supported by our preliminary findings.

To experimentally induce clonus-like epileptic seizures, the animals were individually exposed to 5 mM PTZ, readily dissolved in water. The various parameters of the PTZ seizure-inducing model, such as concentration and the time of exposure, were based on previous reports [24,29,39,55,68,76]. The animals were individually immersed into a 500 mL beaker containing 5 mM PTZ solution, immediately after the first behavioral activity registration.

### 4.4. Behavioral Assessment—Locomotor and Exploratory Behavior

After the anti-epileptic pre-treatment, the animals were carefully placed individually in a rectangular tank (18 × 13 × 15 cm) containing 2.25 L of system water. Their behavioral activity was recorded for a single session of 5 min, by using a digital webcam connected to a computer and placed 40 cm from the testing tank to ensure that the apparatus was within the camera vision range [77]. To investigate the effects of the tested substances on the locomotor and exploratory behavior, we used EthoVision XT version 16 video tracking software (Noldus Information Technology, Wageningen, The Netherlands), which analyzes the tank exploration by adult zebrafish [78]. During the first minute, the animals were habituated to the apparatus and then behavioral activity was analyzed, for a period of 4 min. The locomotion tracking was recorded for each individual and each experimental condition and then the distance moved and the velocity were analyzed as behavioral endpoints [72].

### 4.5. Behavioral Assessment—PTZ-Induced Seizures

All PTZ treatments were videotaped and evaluated later by trained observers. The seizure events were observed for 600 s and scored according to a 5-point scale [31,32] as follows:

1. increased swimming activity and high frequency of opercular movement;
2. burst swimming left and right movements, and erratic movements;
3. circular movements;
4. clonic seizure-like behavior (abnormal whole-body rhythmic muscular contraction);
5. fall to the bottom of the tank, tonic seizure-like behavior (sinking to the bottom of the tank, loss of body posture principally by rigid extension of the body).

The animals were submitted to the PTZ treatment until they reached the last scoring point, which corresponds to the tonic seizure-like behavior in zebrafish, or until 600 s had passed. The occurrence of each seizure stage and the latency to the first behavioral sign of each seizure scoring system stage were analyzed. Experiments were performed in triplicate, on different days in a silent room.

All behavioral data were evaluated by two trained observers in a blind fashion. All necessary precautions were taken to ensure representative behavioral results and to avoid handling stress. Throughout the experiments, the fish were gently transferred between home tanks, beakers, and experimental apparatus. All fish were handled and tested in a similar way and the behaviors were recorded in the same room, which kept the manipulation, water quality, and illumination uniform and constant between trials [68].

### 4.6. Compound Detection and Quantification in Brain Tissue by HPLC and LC-MS

To assess the uptake of the tested substances in the zebrafish brain, the organs were extracted from the skulls and placed into 0.5 mL microcentrifuge tubes. Those from the Cur and MitoCur-treated groups were placed in methanol, while those from the VPA-treated group were placed in PBS, and subsequently homogenized. The sample was further centrifuged and the supernatant was promptly frozen at −150 °C for further analysis [79].

The determinations for VPA were carried out on an Agilent 1220 Infinity II LC high-performance liquid chromatography system (Agilent Technologies, Santa Clara, CA, USA), with an integrated 600-bar pump, autosampler, and diode array detector. MitoCur and Cur were analyzed by a liquid chromatography–mass spectrometry (LC-MS) system, equipped with an Amazon SL mass spectrometer (Bruker Daltonics, Bremen, Germany) and a Dionex UltiMate 3000 high-performance liquid chromatograph (Thermo Fisher Scientific, Waltham, MA, USA), equipped with a quaternary pump (LPG-3400SD chromatograph (Thermo Fisher Scientific, Waltham, MA, USA), maximum pressure of 600 bar, and an autosampler with a column compartment (ACC-3000, Thermo Fisher Scientific, Waltham, MA, USA). Separation was performed on a Phenomenex Kinetex C18 column (Phenomenex, Torrance, CA, USA; 50 × 2.1 mm) (50 × 2.1 mm) with a particle size of 2.6 µm. 

For sample preparation, 100 mg of biological material was mixed with 100 mg of a magnesium sulfate and anhydrous sodium sulfate blend (90%/10% (*w*/*w*)), along with 10 µL of a heptanoic acid stock solution and 10 µL of a quercetin solution, together with 200 µL of water. The sample was homogenized using a vortex mixer for 5 min. Then, 200 µL of acetonitrile was added, and the sample was vortexed for another 5 min. Finally, the samples were centrifuged at 14,000 rpm for 5 min. The separated acetonitrile layer was removed and evaporated to dryness under a nitrogen stream. The residue was reconstituted with 100 µL of mobile phase.

For the HPLC method, the mobile phase consisted of a potassium phosphate buffer (0.04 M, pH 4.0, adjusted with 0.5 M phosphoric acid) and acetonitrile. Mobile Phase A (MPA) was an 80:20 (*v*/*v*) phosphate buffer: acetonitrile mix; while Mobile Phase B (MPB) was a 20:80 (*v*/*v*) phosphate buffer: acetonitrile mix. Elution started with 10% MPB for 1 min, followed by a gradient to 50% MPB in 3 min and maintained for 5 min. Gradient shifted to 70% MPB over 1 min, held for 1 min, then increased to 100% MPB and maintained for 6 min. Initial conditions were restored in 2 min. The flow rate was 1.2 mL/min for 10 min, increased to 1.7 mL/min for 1 min, and held for 6 another minutes. Column temperature was 37 °C, and detection was performed at a 200 nm wavelength.

The mass spectrometer used an ionization voltage of 6 kV, sheath gas pressure at 20 psi, auxiliary gas at 30 psi, and collision gas at 1.5 mtorr. The HESI probe temperature was 450 °C. Detection for Cur and quercetin was carried out in negative mode using *m*/*z* 367.0 → *m*/*z* 216.95 for Cur and *m*/*z* 301.0 → *m*/*z* 178.89 for quercetin. The detection of MitoCur was performed in positive mode, using the transition *m*/*z* 487.3 → *m*/*z* 303.1.

Regarding the LC-MS conditions, the mobile phase consisted of a 10 mM ammonium formate buffer (MPA) and acetonitrile (MPB). A gradient elution started with 10% MPB for 1 min, followed by a gradient to 50% MPB over 3 min, then to 90% MPB in 1 min and held for another minute. Conditions shifted back to 50% MPB, with re-equilibration to initial conditions in 2 min.

Calibration of VPA included 60.0, 40.0, and 20.0 ppm concentrations and 25 ppm for internal standard (heptanoic acid). The calibrations of Cur and MitoCur included 5.0, 50 and 200 ppb, with 100 ppb for the internal standard.

### 4.7. Data Analysis

Statistical analysis was performed using GraphPad Prism version 10.3.1 for Windows, GraphPad Software, Boston, MA, USA, www.graphpad.com.

## 5. Conclusions

In conclusion, this study highlights the anticonvulsant properties of MitoCur, investigated for the first time in a zebrafish model of PTZ-induced seizures. Its observed efficacy compared to standard Cur, combined with its mitochondrial-targeted action, positions MitoCur as a potential candidate for the management of epilepsy, particularly in cases involving mitochondrial dysfunction. These findings lay the groundwork for future research into innovative therapeutic strategies that could address the limitations of current antiepileptic treatments. Further investigations, particularly in mammalian models and clinical settings, are essential to validate these results and fully understand the therapeutic potential of mitochondrial-targeted approaches in epilepsy management.

## Data Availability

The raw data supporting the conclusions of this article will be made available by the authors on request.

## Supplementary Materials

The following supporting information can be downloaded at: https://www.mdpi.com/article/10.3390/ph17121611/s1, Figure S1. Pretreatment; Video S1. Behavioral tests—distance and velocity; Video S2. Latency score 4 and 5 during PTZ exposure.
