# Supplementary figures and images for: Anti-Epileptic Activity of Mitocurcumin in a Zebrafish–Pentylenetetrazole (PTZ) Epilepsy Model
Source: Pharmaceuticals (Basel). 2024 Nov 29;17(12):1611. doi: 10.3390/ph17121611 (PMC11678555; doi:10.3390/ph17121611)

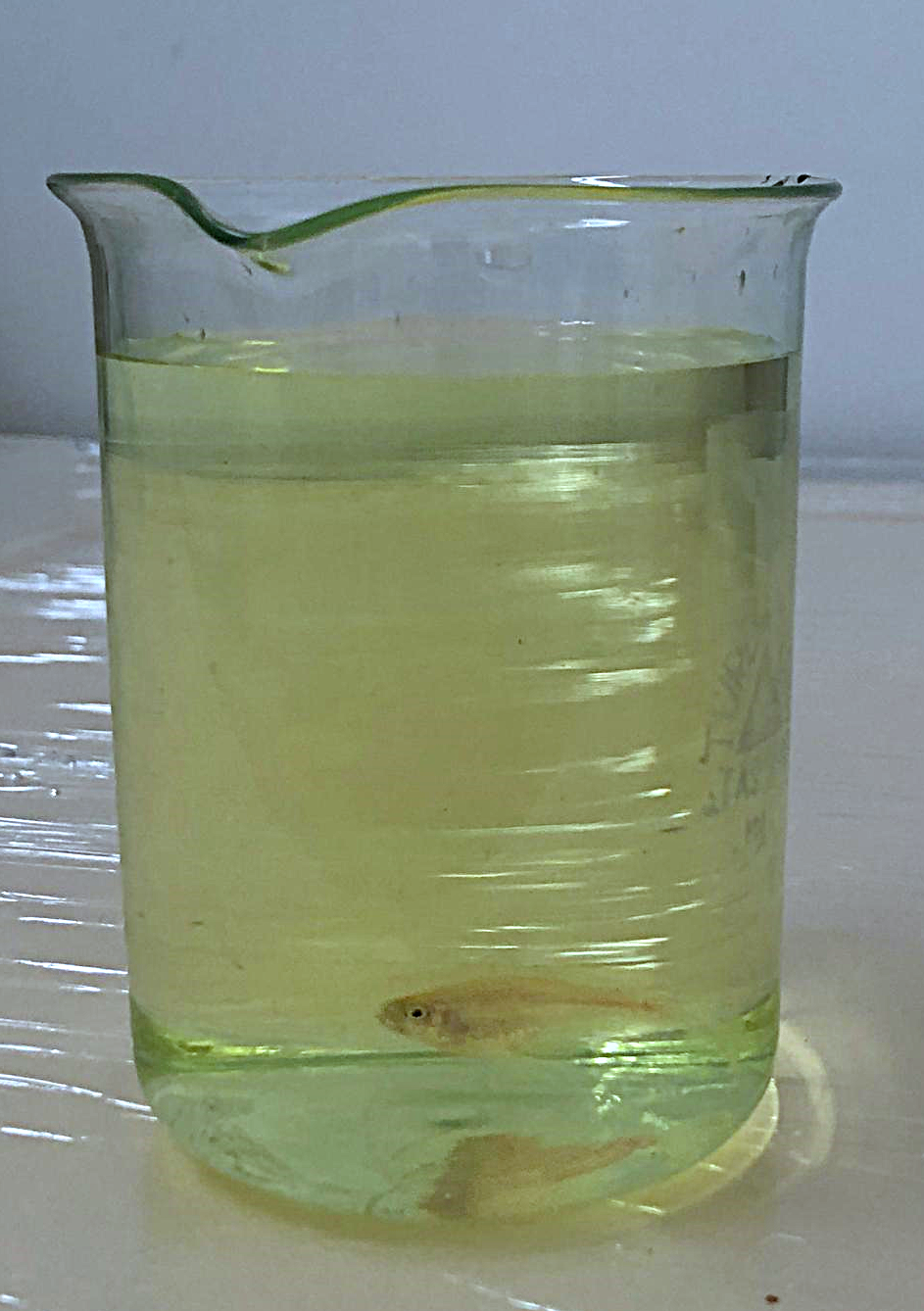

Supplement: Supplementary file 1 [file pharmaceuticals-17-01611-s001.zip › Figure-S1-Pretreatment.tif]
